# Adversarial Gaussian Denoiser for Multiple-Level Image Denoising

**DOI:** 10.3390/s21092998

**Published:** 2021-04-24

**Authors:** Aamir Khan, Weidong Jin, Amir Haider, MuhibUr Rahman, Desheng Wang

**Affiliations:** 1 School of Electrical Engineering, Southwest Jiaotong University, Chengdu 611756, China; aamir@my.swjtu.edu.cn (A.K.); wds@my.swjtu.edu.cn (D.W.); 2 China-ASEAN International Joint Laboratory of Integrated Transport, Nanning University, Nanning 530000, China; 3 Department of Intelligent Mechatronics Engineering, Sejong University, Seoul 05006, Korea; amirhaider@sejong.ac.kr; 4 Department of Electrical Engineering, Polytechnique Montreal, Montreal, QC H3T 1J4, Canada; muhibur.rahman@polymtl.ca

**Keywords:** image denoising, residual learning image denoising (RLID), direct image denoising (DID), convolutional neural networks (CNNs), generative adversarial network (GAN)

## Abstract

Image denoising is a challenging task that is essential in numerous computer vision and image processing problems. This study proposes and applies a generative adversarial network-based image denoising training architecture to multiple-level Gaussian image denoising tasks. Convolutional neural network-based denoising approaches come across a blurriness issue that produces denoised images blurry on texture details. To resolve the blurriness issue, we first performed a theoretical study of the cause of the problem. Subsequently, we proposed an adversarial Gaussian denoiser network, which uses the generative adversarial network-based adversarial learning process for image denoising tasks. This framework resolves the blurriness problem by encouraging the denoiser network to find the distribution of sharp noise-free images instead of blurry images. Experimental results demonstrate that the proposed framework can effectively resolve the blurriness problem and achieve significant denoising efficiency than the state-of-the-art denoising methods.

## 1. Introduction

**Figure 1:**
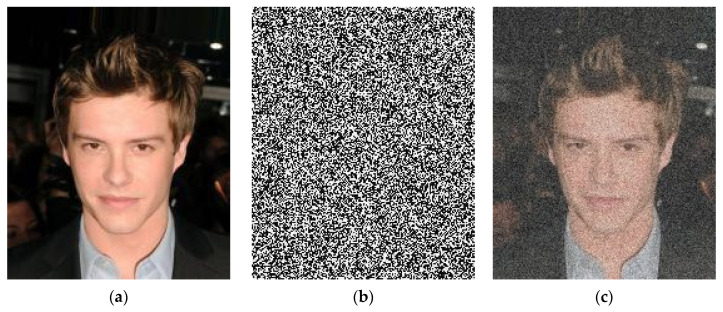
Sample of degraded image. (**a**) presents an original image y, (**b**) demonstrates the AWGN image n, and (**c**) shows the resultant image x=y+n.

Image denoising has become a popular topic in the field of low-level and high-level vision problems, but it remains an essential and difficult task. Due to the image sensing process, the various inevitable noises reduce the visual quality of an image. The elimination process of noise from the observed image is essential in numerous computer vision and image processing issues [1,2,3,4]. Image denoising plays an important role in the fields of computer vision and image processing with diverse applications like medical imaging, remote sensing, military and surveillance, robotics, and artificial intelligence, where obtaining the original image content is crucial for strong performance [5]. The image degradation concept can be described mathematically as x=y+n, where x is the degraded form of the original image y, and n is the added noise, generally referred as additive white Gaussian noise (AWGN) as shown in Figure 1. Methods of image denoising concentrate on restoring the denoised image y from its cross ponding noisy image x through eliminating or reducing noise n. To date, a denoising method that has given very satisfactory results is that based on first generation [6,7] and second generation wavelets such as curvelets [8,9] or contourlets [10,11]. These methods carry out a multiresolution analysis [12] or multiscale analysis for denoising an additive white and Gaussian noise. The most targeted applications are in the field of medical imaging [13,14]. Image denoising techniques can be divided into two main groups: model-based and discriminative learning-based image denoising techniques. Model-based approaches can tackle image denoising issues by varying noise levels; however, the noise levels must be identified in advance. Despite having some weaknesses, they have demonstrated good results. A significant obstacle to model-based denoising approaches is that they typically take advantage of handcrafted image priors (e.g., priors of sparsity [15,16] and priors of non-local self-similarity (NSS) [17,18,19,20]), which are incapable of characterizing complicated image structures. Another drawback is that the complicated optimization method being conducted during inference is time-consuming, thus taking a considerably long denoising time. They also cannot eliminate variant noises in spatial terms. Block-matching and 3D filtering (BM3D) [18] is one of the most famous and state-of-the-art techniques among the many NSS models.

Discriminative learning-based approaches have been adopted to resolve the drawbacks of model-based approaches and address the disadvantages above-mentioned. Discriminative denoising techniques aim to learn a noise model from a specified collection of distorted training data and corresponding clean image pairs in the training process. These techniques do not require any adaptive refinement during the test interval, leading to a faster denoising speed, which is the main benefit. In particular, the convolutional neural network (CNNs) based approaches are now the most famous in the discriminatory learning techniques because CNNs has characteristics such as sparse relation and weight sharing. These properties are simpler to train the CNN-based models and more comfortable to prevent the issue of overfitting.

In theory, approaches based on CNNs can also be divided into three groups. The first group includes the prior information-based approaches. These procedures train a denoiser network as per certain statistical rules. For example, NOISE2NOISE [21] uses several different pairs of independently distorted images of identical scenes to train its denoising network. This approach is built on the statistical rule that a network will be directed by the L2 loss to find the mean solution for all possible solutions. In addition, NOISE2VOID [22] provides a simplified approach in which only several single noisy images of various scenes are used to train the denoising network. The average of the target noisy pixel’s surrounding pixels is taken as the corresponding clean pixel as per the image’s local resemblance. This method can overcome the need to train a denoising network for many pairs of images. Nevertheless, their denoising result was constrained by the prior information used.

The second group is a simple denoising method, which split up noise from the given distorted input image [23,24,25]. Feedforward denoising (Dn)-CNN [23] harnesses the deep convolutional neural networks’ achievement on image denoising tasks and is a popular traditional approach due to the good denoising efficiency. Instead of learning direct clean targeted images, Dn-CNN maps residual images (noise images) and produces target images by subtracting residual images from the input images. Through CNN model parameters, Dn-CNN finds the mapping relationship between the noisy and targeted clean images. Distinct loss functions and different motivations have developed CNN models [26,27]. These models use reconstruction or pixel-wise losses [26,28,29,30] to produce output images, being the most popular methods. For example, the least-squares or least absolute losses in pixel space are utilized to calculate the targeted and constructed images’ variance. The pixel-wise calculation can produce reasonable images. Though, during some instances, these loss functions mostly catch low frequency rather than high frequency elements of images, resulting in certain critical performance drawbacks (e.g., image artifacts and image blurring) [31,32].

The third group is the generative methods that reduce noise through two stages: modeling of noise and supervised denoising. For supervised denoising, the noise modeling process first designs real-world noise utilizing real-world residual images and then produces several clean noisy image pairs. The generated image pairs are used to train a denoising network and find the mapping relationship in the supervised denoising process. For example, GCBD [33] uses generative adversarial networks (GANs) [34] that design real-world noise and produce several pairs of clean noisy images by the addition of their created noise with a single clean image dataset. GANs have shown remarkable outcomes in image generation tasks. GANs, presented by Goodfellow et al. [34], consists of a generator network and a discriminator network, aimed at modeling the distribution of the real images via refining created samples that are very close to the actual images. The GAN-based model produces more realistic and sharper images than CNN-based models [35,36,37], which is a substantial benefit of using it. Image denoising tasks based on paired images could be formulated as a paired image-to-image translation task. GANs and conditional GAN (cGANs) [38] procedures had proven to be the traditional method for image-to-image translation problems [35,39]. Pix2pix-cGAN [35], based on cGAN, has become a popular method to resolve the paired image-to-image transformation problems and map the distribution of the actual images conditioned on the input images [37,40,41,42,43]. In the literature, most of the methods used residual learning for image denoising tasks. For example, based on Wasserstein generative adversarial networks (WGAN) [44], Chen et al. [45] proposed an image denoising training scheme and used residual learning for the generator network. In the residual learning image denoising methods, the network learns the residual image (noise image) first and subtracts the residual image from the input image to get the clean image. This method is beneficial for low levels of noise. Nevertheless, this method does not simplify well enough for numerous noise levels and generates over-smoothed results for the higher noise level and giving up the fine image details. Hence, the visual performance of the produced images is not pleasing [46].

We proposed the adversarial Gaussian denoiser network (AGDN) using adversarial and reconstruction losses to overcome image artifacts for the high levels of image denoising tasks that construct the sharp and target-oriented images. Instead of using the skip-connections, the proposed model uses residual blocks [47] between the encoder and decoder networks for the deep sparse understanding of the input images.

The remaining research is as follows. In Section 2, the previous image denoising research is presented in detail. Section 3 explains the proposed methodology, objective function, and network architecture. The experiments, datasets, model parameters, results, the study of various loss functions, and network configurations with different methods are discussed in Section 4. Our conclusions and future studies are discussed in Section 5.

## 2. Related Work

Discriminative learning-based techniques have become quite common due to sensible, practical, remarkable results, and a short testing time. In this section, we describe in detail the three types of discriminatory learning-based approaches.

This form of denoising technique explicitly learns a prior model. In model-based approaches, the model first learns the image prior and then implements adaptive refinement in the testing phase. However, discriminative learning methods [48,49,50,51] aim to learn by minimizing a predefined loss function during the training phase, and there is no optimization required in the testing time. Barbu [52] introduced the active Markov random-field (MRF) architecture by merging MRF with a faster testing process for image denoising. A non-local range (NLR)-MRF was introduced by Sun and Tappen [53] to boost the performance of maximum a posteriori (MAP) by parameters optimizing a continuous-valued MRF during the testing phase. Both algorithms were trained by the minimization of the objective function through gradient-based learning techniques. While the above methods can discriminatively pick up the prior parameters, their inference attributes are phase-invariant, subsequently less simplification control for different noise levels.

Schmidt and Roth [48] proposed the cascade of shrinkage fields (CSF) approach and the trainable nonlinear reaction-diffusion (TNRD) model proposed by Chen et al. [49] provide some illustrative examples of discriminative-based learning models. CSF merges the random field-based scheme using the half-quadratic optimization architecture and the process of optimization in the single learning algorithm. TNRD finds an improved expert’s image prior field with gradient-descent inference through the constant number of iterations. TNRD utilizes additional filters by bigger kernel sizes, dynamic punishments in random forms, and changing each iteration parameter. CSF and TNRD demonstrated good results in computational performance and denoising quality. However, their efficiency is limited to specified categories of prior because of their limitation in capturing the complete image structure. Moreover, with many handcrafted parameters, TNRD and CSF are well-tuned to some amount of noise. Subsequently, they do not apply to multiple image denoising tasks.

Recently, due to CNN’s significant success in computer vision, image denoising work has attracted wide attention and made much improvement by utilizing CNN models. Simple discriminative learning models discover mapping functions and predict the image prior implicitly by using CNN’s strength. Jain and Seung [54] introduced a scheme that used the five-layer CNN of sigmoid non-linearity. Mao et al. [55] introduced a full convolution layer encoder-decoder framework with synchronous skip connections aimed at the image reconstruction tasks. Xie et al. [56] introduced a denoising algorithm that combines denoising auto-encoder and sparse coding by a training method that applies a pre-trained denoising auto-encoder aimed at image denoising tasks. However, those initial denoising approaches [54,55,56] failed to cope with the benchmark denoising methods.

Zhang et al. [23] introduced the Dn-CNN for image denoising tasks. The Dn-CNN is a discriminative-based learning method that discovers a relationship between the given distorted image and targeted clean image by utilizing the CNN model’s parameters and demonstrated impressive denoising results. These models were trained to learn the residual images between noisy images and noise-free images. They utilized batch-normalization methods to boost performance and speed up the learning procedure. Zhang et al. [57] introduced deep denoising networks that offer a trade-off between the inference time and the output. They used a dilated convolution layer [58] to have a model with a larger receptive area.

Moreover, Zhang et al. [24] introduced a flexible and faster denoising (FFD)-CNN-based image denoising approach (FFDNet) to resolve several noise levels and spatially different noises with a single model. This method receives a configurable noise-level map as the extra input with a down-sampled distorted image. It utilizes feedforward CNNs to construct the targeted clean image. Rather than using the dilated convolution method to raise the receptive fields, it works with downsampled sub-images that help attain the larger receptive fields without creating any image artifacts. Furthermore, the downsampling process significantly reduces the testing time.

In GAN-based models, the generator network is similar to the CNN’s encoder-decoder structure. The deep-CNNs suffer from a disappearing gradient issue during the training process. Consequently, many previous studies [45,46,59] have utilized skip-connections in a generator network to easily allow a gradient to earlier network layers. Unfortunately, such skip-connections bring unwanted data straight from the input images to the constructed images, reducing the constructing images’ visual quality. The denoising tasks for the low level of noise can benefit from any of the above-mentioned methods. However, these denoising methods sacrifice adequate image information when dealing with higher noise levels, resulting in image artifacts and over-smoothed images. Consequently, the produced images have poor visual quality. We must factor the following information into the denoising model’s optimization process to construct target-oriented and visually pleasing images.

- The concept of perfect mapping targeted noise-free images should not be influenced by the appearance of given noisy images, which must be the foundation of any denoiser network.
- Rather than depending solely on output qualitative metric values, the graphic visual quality factor of generated images must be considered during the optimization process. This principle ensures that the produced images are realistic and visually pleasing.

Based on the above criterion, we proposed the adversarial Gaussian denoiser network (AGDN) for all levels of image denoising tasks. The AGDN contains a denoiser network and the discriminative network. The denoiser network transforms the noisy input images into noise-free targeted images, whereas the discriminator network distinguishes between the fake and real images. This study employs the pixel reconstruction L1 loss and adversarial losses in the loss function. We used the traditional L1 loss to push constructed images to stay close to clean targeted images. In the meantime, we utilized the adversarial loss to calculate the constructed image distribution, that is, to push the constructed distribution to converge into clean targeted distribution, which usually results in less blurry, sharper, and pleasing images. This study’s contributions are as follows:

- This work presents a novel approach for all the levels of Gaussian image denoising tasks. It uses the direct image denoising method via an encoder-decoder denoiser trained by adversarial and reconstruction losses.
- This study introduces an optimized technique based on conditional GAN (cGANs) architecture for image denoising tasks.
- We deeply analyzed the traditional two methods (i.e., residual learning image denoising method and direct image denoising method) for image denoising tasks on the denoiser network’s two different primary configurations. The results demonstrate that the proposed method is an agreeable alternative for image denoising tasks.
- We also achieved quantitative and qualitative results using AGDN, which expresses that the proposed method generates better results than the state-of-the-art methods.

Table 1 presents the comparison among the proposed AGDN and current state-of-the-art methods.

## 3. Methodology

We proposed an image denoising training scheme by merging adversarial losses with reconstruction losses and learn the clean target images directly instead of residual images to resolve the blurriness and image artifacts issue. Additionally, we fine-tune the training specifics of pix2pix-cGAN to make it appropriate for image denoising tasks.

In this study, we used two kinds of pair training examples, that is, a set of noisy input images ${\left\{x_{i}\right\}}_{i=1}^{N}\in X$, and a set of clean target images ${\left\{y_{i}\right\}}_{i=1}^{N}\in Y$. The denoiser network V was trained so that the constructed noise-free images V(x) were similar to the actual clean target images, and we simultaneously trained the discriminator network, D, to differentiate the fake constructed noise-free photos from the actual clean photos. The denoiser learns the transformation from a noisy-domain to a clean real-domain through minimizing the adversarial losses, attempting to trick the discriminative network. The denoising network contains an encoder network En, residual blocks layer R, and the decoder network De. The encoder includes a set of downsampling convolutional layers that transform a noisy image into some feature domains En(x). Later, these feature domains, En(x), feed to the residual blocks [47]. The output feature maps of residual blocks, R(En(x)), becomes the input of the decoder network De. At that point, a series of up sampling transposed convolution layers decode the transformed feature maps into fake constructed clean image V(x). The output of the denoising network is described in Equation (1).

(1) V(x)=De(R(En(x)))

**Figure 2:**
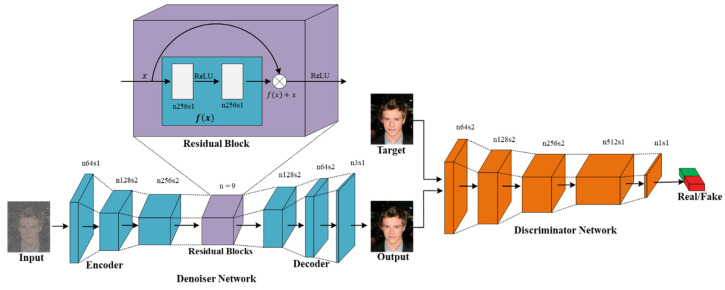
AGDN framework. AGDN consists of the denoiser, V, and the discriminator, D. The denoiser, V, aims to construct noise-free images from the given noisy images. It consists of the encoder-decoder configuration with three down-sampling convolution stride-1 and stride-2 layers, nine residual blocks, two up-sampling transposed convolution layers of stride-2, and one convolutional layer of stride-1. The discriminator, D, includes the convolutional batch-normalization leaky ReLU layers, and the output of D is utilized to differentiate the constructed images from the real images.

Figure 2 illustrates the entire network framework known as the adversarial Gaussian denoiser network (AGDN).

### 3.1. Objective Function

The generator and discriminator networks were trained by GAN losses [34]. The GAN losses constitute two parts: the first one is termed as the mini-max GAN loss, and the second one as the non-saturating GAN loss. Minimax GAN loss refers to the mini-max simultaneous optimization of the discriminator and generator models. The non-saturating GAN loss is a modification to the generator loss to overcome the saturation problem by maximizing the log of the discriminator probabilities for generated images. The generator network tries to construct an image that should be similar to the image present in the targeted domain Y, whereas the discriminator network aims to distinguish between the constructed (i.e., fake) image and targeted (i.e., real) image. Adversarial training is similar to a two-player mini-max game where the discriminator is trained for maximizing the probability of correctly classifying the fake images (i.e., coming from the generator and the real images, i.e., coming from targeted images), while the generator network is trained to minimize the probability of correctly classifying the constructed image by the discriminator network. Equation (2) expresses the mini-max game.

(2) $$\underset{G}{\min}\;\underset{D}{\max}\;E_{y\in Y}\left[\log\left(D\left(y\right)\right)\right]+E_{x\in X}\left[\log\left(1-D\left(G\left(x\right)\right)\right)\right]$$

GAN-based methods have shown a significant potential to understand generative models, especially for artificial image generation works [44,60,61]. Therefore, as a result, we used the GAN-based learning method to solve image denoising problems. The denoiser network V was utilized to construct a noise-free clean image, V(x), against corresponding noisy image, x∈X, as shown in Figure 2. Meanwhile, each noisy input image $x_{i}$ has a corresponding noise-free target image $y_{i}$. We presumed that all noise-free targeted images, y, belonged to the distribution y∈Y, and the constructed noise-free images, V(x), were encouraged to obtain the same distribution as the noise-free targeted images y (i.e., V(x)~Y). In addition, to achieve the adversarial learning method, a discriminator network, D, is introduced, and the adversarial objective function can be described as follows:

(3) $$\underset{V}{\min}\;\underset{D}{\max}\;F\left(V,D\right)=E_{y\in Y}\left[\log\left(D\left(y\right)\right)\right]+E_{x\in X}\left[\log\left(1-D\left(V\left(x\right)\right)\right)\right]$$

As discussed in [62], we utilized the least square loss (LSGAN), which provides a smooth and non-saturated gradient for the D network. Adversarial loss, ${ℒ}_{GAN}\left(V,D\right)$, is formulated as follows:

(4) $${ℒ}_{GAN}\left(V,D\right)=E_{y\in Y}\;\left[{\left(D\left(y\right)-1\right)}^{2}\right]+E_{x\in X}\left[D{\left(V\left(x\right)\right)}^{2}\right]$$

The adversarial losses respond to the numerical calculation to penalize the difference between the noise-free constructed and noise-free targeted image distributions.

The traditional GAN architecture is unstable since it needs to train two opposing neural networks. One cause of instability, according to [63], is that there are multiple solutions during the generator network training. Previous studies have revealed that it is helpful to merge the GAN objective function with other conventional losses like L2 loss [64], so that the discriminator’s function remains unchanged, like in Equation (4). However, the generator’s function is to deceive the discriminator network and generate images nearer to the target images due to L2 loss. We utilized the L1 loss in the proposed method instead of the L2 loss because the L1 loss encourages less blurriness. The L1 loss can be expressed as follows:

(5) $${ℒ}_{L1}\left(V\right)=E_{x,y}\left[\|y-V{\left(x\right)\|}_{1}\right]$$

The adversarial losses assist the denoiser network in protecting the blurriness effect of L1 loss and remaining near the target images. The total objective function of the denoiser network can be described as:

(6) $${ℒ}_{V_{T}}=\varphi_{v}{ℒ}_{GAN}\left(V\right)+\varphi_{L1}{ℒ}_{L1}\left(V\right)$$

where ${ℒ}_{V_{T}}$ denotes the total denoiser network loss, that is, the summation of the denoiser’s adversarial loss, ${ℒ}_{GAN}\left(V\right)$, and L1 reconstruction loss, ${ℒ}_{L1}\left(V\right)$.

### 3.2. Network Architecture

Figure 2 illustrates the proposed framework contains two CNN networks, that is, the denoiser network, V, and the discriminator network, D.

Many solutions [45,46,59] to denoising problems utilized skip-connection in the denoiser network, transporting the data directly from the input to the output through the network for resolving the disappearing gradient issue. On one hand, skip-connections help resolve the vanishing gradient issue. These skip-connections carry unwanted data from the noisy input through all the decoder network layers and critically influence the quality of the constructed images for image denoising tasks. To prevent unwanted information flow and produce visually pleasing results, we utilized the ResNet [47] architecture, similar to Johnson et al. [65], through an encoder-decoder configuration rather than using skip-connections, as shown in Figure 2. Our denoiser network consists of three down-sampling convolution layers of stride-1 and stride-2, nine residual block layers, two up-sampling transposed convolution layers of stride-2, and one convolutional layer of stride-1. It utilizes instance normalization [66]; for detailed specifications, see Table 2 and Table 3.

In this study, we utilized the Markovian 70 × 70 PatchGANs [31,35,67] in the discriminator network, D, to examine whether the overlapping 70 × 70 image’ patches are fake or real. Patch-level discriminators have less parameters than the full-image discriminators and can work on images of any scale in a fully convolutional fashion [35]; for detailed specifications, see Table 4.

## 4. Experiments and Results

First, we address the dataset, the training parameters, and the proposed model details in this section. We compare the AGDN with the traditional techniques and the existing state-of-the-art approaches. We also analyze the experiment details and quality metrics used to evaluate the proposed scheme.

### 4.1. Dataset

This study used the Partial-CelebA dataset [68] and DIV2K dataset [69]. We randomly selected 1500 and 800 images from the Partial-CelebA and DIV2K datasets, respectively, to conduct the training in our experiments for each noise level. Additionally, 500 and 100 test images were randomly selected from the Partial-CelebA and DIV2K datasets, respectively, to do the cross validation of the proposed model for each noise level. To make the pair of noisy and target images, we created distorted images from the dataset images via inducing AWGN as

$$x_{i}=y_{i}+n{\left(\sigma \right)}_{i}\;$$

where y is the target original image and x is the corresponding noisy image produced via AWGN; and n(σ), with standard deviation σ. The number of experiments was undertaken on four different noise levels by changing the numerical value of σ as 5, 25, 50, and 100 for both datasets.

### 4.2. Parameter and Model Details

In this sub-section, we describe the parameter and the model details. For the model’s training stabilization, we substituted the metric of negative-log-likelihood with the least-square-loss [62] in the case of GAN loss (${ℒ}_{GAN}$). The least-square-loss works more consistently during training and generates good results, which are close to the target images. In particular, for ${ℒ}_{GAN}\left(V,D\right)$, the V, was trained to minimize $E_{x\sim p_{data}\left(x\right)}\left[{\left(D\left(V\left(x\right)\right)-1\right)}^{2}\right]$ and the D, was trained to minimize $E_{y\sim p_{data}\left(y\right)}\left[{\left(D\left(y\right)-1\right)}^{2}\right]+E_{x\sim p_{data}\left(x\right)}\left[(D{\left(V\left(x\right)\right)}^{2}\right]$. Moreover, when optimizing D, here the discriminator’s criterion was divided by 2, which slows down the learning-rate of D compared to V. We used the Adam optimizer [70] with a learning rate of α=0.0002, β1=0.5, and a minibatch stochastic gradient decent (SGD). We used the relu non-linear activation function, along with the slope of 0.2, in the denoiser network, V, excluding the final layer utilized tanh activation. For all the experiments, the batch-size was fixed to 1. The loss function parameters for training were set to $\varphi_{g}=1$ and $\varphi_{L1}=10$ in Equation (6).

### 4.3. Evaluation Criteria

We used qualitative and quantitative tests to assess the quality of the resulting images for performance validation of the image denoising works. We specifically present the target and resultant images for the qualitative evaluation. We used quantitative measurements including peak signal to noise ratio (PSNR), structural similarity index measurement (SSIM) [71], visual information fidelity (VIF) [72], and universal quality index (UQI) [73] on test images to evaluate the output of different methods. Such quantitative measurement evaluation was built on the images’ luminance channel. The Fréchet inception distance (FID) score [74] calculates the gap between the actual distribution and the constructed distribution.

### 4.4. Loss Functions Ablation Study

**Figure 3:**
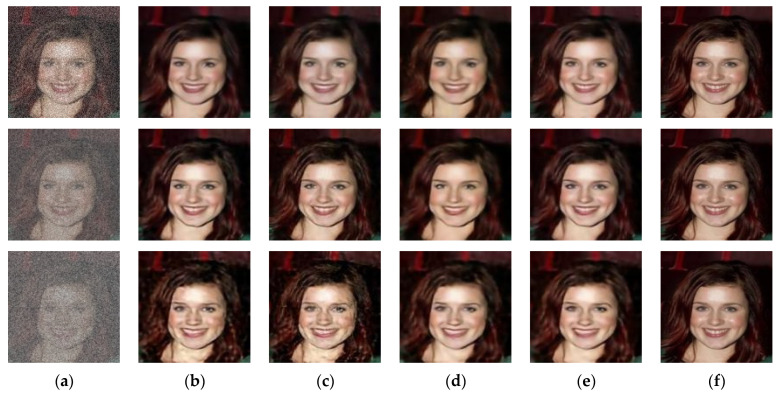
Results of different loss functions that construct different noise-free images from corresponding noisy images. The first row results are the noise level of sigma 25. The second row results are the noise level of sigma 50. The third row results are the noise level of sigma 100. (**a**) input noisy image, (**b**) result of L2 alone, (**c**) result of L2 and adversarial loss, (**d**) result of L1 alone, (**e**) result of the proposed AGDN loss function, and (**f**) target image.

We trained our model on different loss functions to check their impact on the higher noise levels by setting the sigma value to 25, 50, and 100. We ran tests to compare the effect of different loss functions. Figure 3 illustrates the qualitative performance of the different loss functions mentioned below on a higher noise level.

- L2 loss alone causes the reconstruction of noise-free images with many image artifacts.
- L2 with adversarial loss guides to sharper outputs; however, it brings more visual artifacts.
- L1 alone produces sensible results, but their resultant images were not much sharper.
- The proposed loss function’s performance illustrates the significant improvement and constructs a sharper quality and similar images to the targeted images.

Table 5, Table 6 and Table 7 quantitatively compare the cases above-mentioned by utilizing the PSNR, SSIM, UQI, VIF, and FID metrics on the higher-levels of noisy images (i.e., sigma 25, sigma 50, and sigma 100, respectively). Table 5 shows that L2 loss alone achieves good scores than L2 loss with adversarial loss and L1 loss alone in PSNR, SSIM, UQI, VIF, and FID. Figure 3 shows that L1 loss alone produces blurry results and the second row of Figure 3 illustrates that the L2 loss alone and L2 loss with adversarial loss produced image artifacts. The proposed loss function overcame the blurriness issue of L1 loss alone. Table 5, Table 6 and Table 7 and Figure 3 demonstrate that the proposed method achieved the best possible score in PSNR, SSIM, UQI, VIF, and FID scores, pointing out that the results were more similar to the targeted output, had a recognizable structure, and were visually pleasing.

### 4.5. Analysis of Residual Learning and Direct Image Denoising Training on Different Configurations

**Figure 4:**
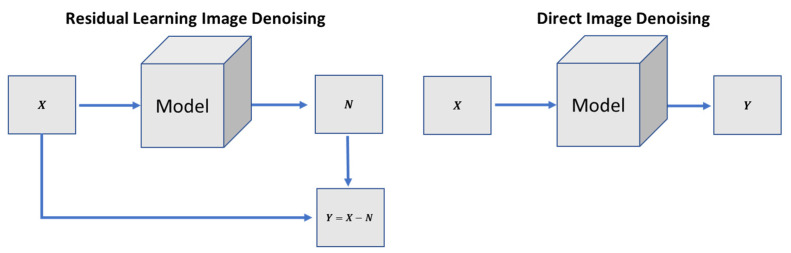
Different approaches for image denoising tasks.

**Figure 5:**
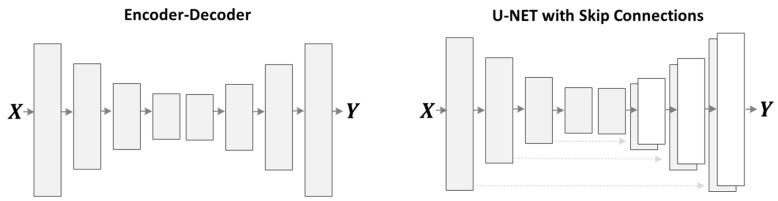
The different network structures for the image generation network. The left one is the encoder-decoder structure, where first the image is encoded to some latent space, and then decoded for target image reconstruction. The right one is the U-NET structure, where the encoder and decoder are connected with skip-connections.

In the residual learning image denoising (RLID) method, the network learns the residual image (noise image) first. It then subtracts the residual image from the input image to get a noise-free target image. In the direct image denoising (DID) method, the model directly tries to learn the noise-free target image, as shown in Figure 4. We have trained both methods and the primary two configurations of the image generating network as shown in Figure 5 on multiple noise levels for image denoising tasks. We conducted tests to compare both methods on two primary configurations of the image generating network. Table 8 compares the cases above-mentioned quantitatively by utilizing the PSNR, SSIM, UQI, VIF, and FID metrics on low and high levels of denoising tasks.

**Figure 6:**
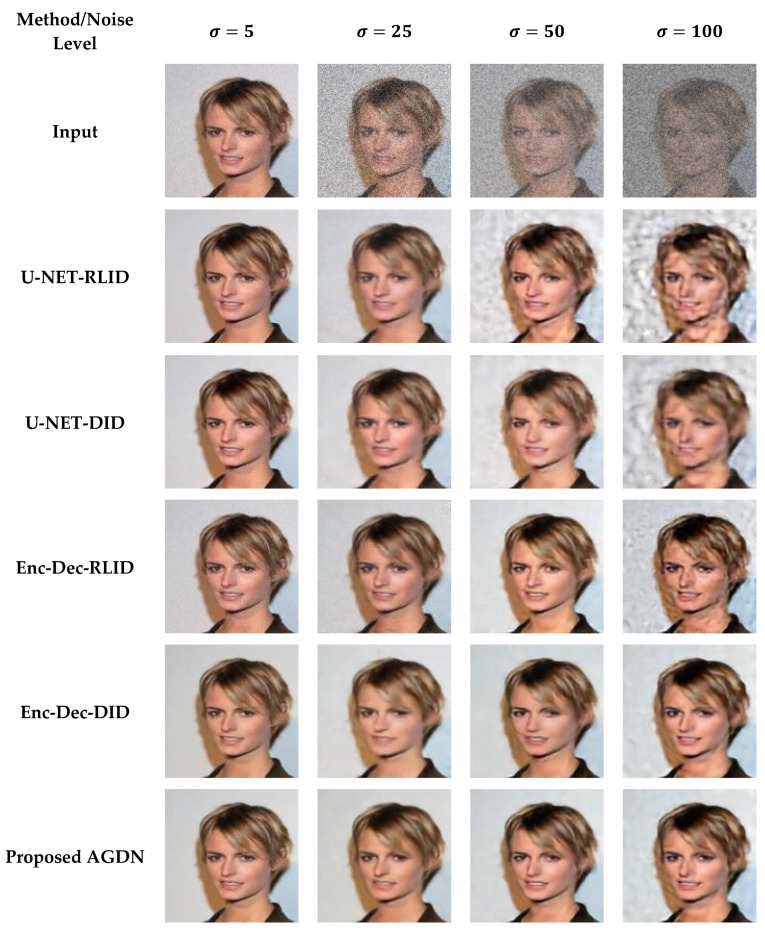
Sample results of the image denoising task using different methods and network structures. The first row is the input images of different noise levels. The second row shows the results produced by the RLID method using the U-NET structure. The third row presents the U-NET structure results via the DID method. The fourth row shows the encoder-decoder structure results using the RLID method. The fifth row presents the encoder-decoder structure results via the DID method. The sixth row demonstrates the results of the proposed AGDN.

Figure 6 shows that the U-NET structure with the DID method achieved better results than the RLID method. Furthermore, the encoder-decoder structure using the RLID method outperformed the U-NET structure using both methods (i.e., RLID and DID methods). However, it did not perform well on higher noise level images (e.g., σ=100) and produces image artifacts. The DID method using the encoder-decoder structure did not produce image artifacts compared to the RLID method, but constructed the blurry output images. To overcome the blurriness issue, we introduced adversarial loss to the proposed method’s objective loss function. The last row of Figure 6 shows the sharp, pleasing, and consistently excellent results of the proposed method for low and higher noise levels.

We observed from Figure 6 that the RLID method produces visual artifacts. It failed to produce pleasing images for the higher noise level, which shows that the model learns from direct clean targeted images easier than learning the noise image first and then constructing the target image. The U-NET structure failed to reconstruct the target images for high-level noisy input images because the skip-connections carry unwanted details from the input images, severely influencing the output images, causing distorted results, and failing to construct the clean target images. However, the DID using an encoder-decoder structure’s network produces good and less image artifacts for low and higher noise levels.

### 4.6. Comparison with Baseline Methods

For evaluation purposes, we compared our proposed method with the latest state-of-the-art image denoising approaches. The compared techniques included Dn-CNN [23], FFDNet [24], perceptually inspired denoising method [46], and ID-MSE-WGAN [45]. The Dn-CNN and ID-MSE-WGAN predicted the noise first and then constructed the target images by subtracting that learned noise from the input images. These methods construct reasonable images for low noise levels; however, for higher noise levels, these methods produce image artifacts. To capture larger receptive fields, the FFDNet utilizes downsampled sub-images. However, downsampling of images can cause the loss of important information in the images. The perceptually inspired denoising method [46] uses skip-connections in the encoder-decoder network for securing larger receptive fields. However, the skip-connections cause unwanted information flow from the encoder layers to the decoder layers, producing unpleasant images [32].

#### 4.6.1. Partial-CelebA Dataset

**Figure 7:**
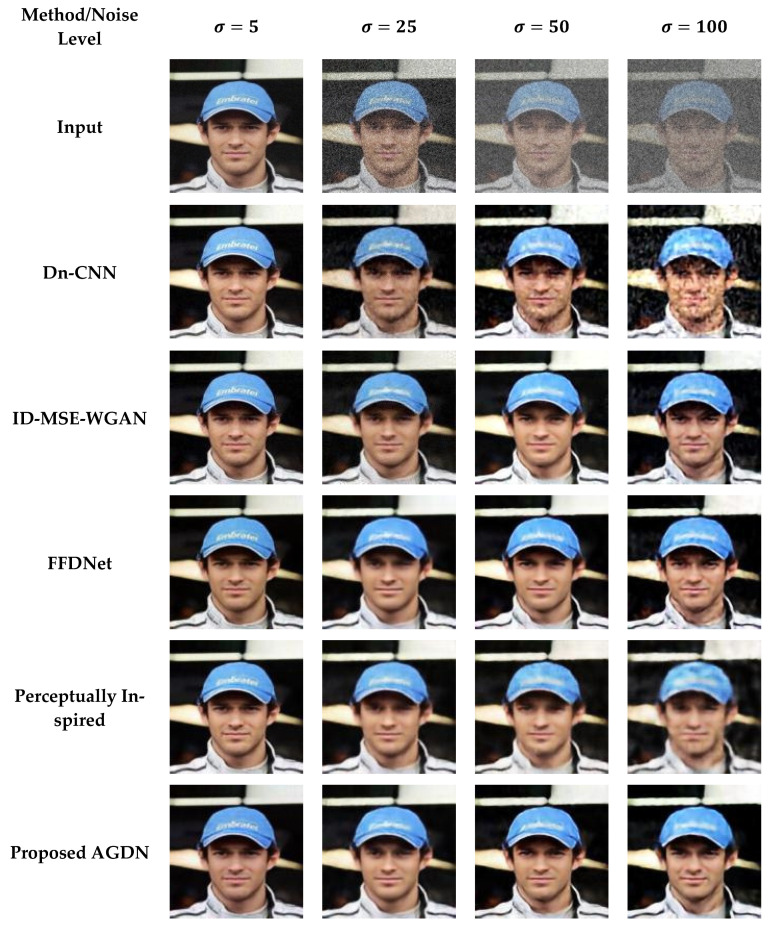
First sample results of image denoising tasks on the Partial-CelebA dataset. The First to last column images generated by the noise level of sigma 5, 25, 50, and 100, respectively. First-row shows input images, Second-row to the last-row presents the results of Dn-CNN, ID-MSE-WGAN, FFDNet, the perceptually inspired method, and the proposed AGDN, respectively.

**Figure 8:**
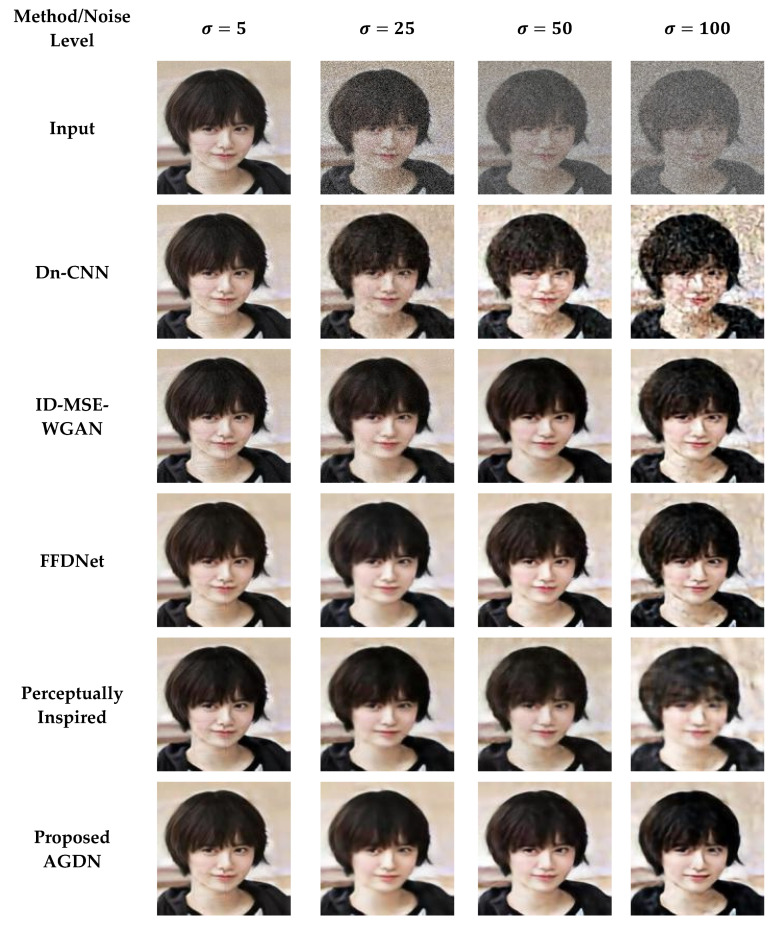
Second sample results of image denoising tasks on the Partial-CelebA dataset. The first to last column images were generated by the noise level of sigma 5, 25, 50, and 100, respectively. First-row shows the input images, second-row to the last-row presents the results of the Dn-CNN, ID-MSE-WGAN, FFDNet, the perceptually inspired method, and the proposed AGDN, respectively.

**Figure 9:**
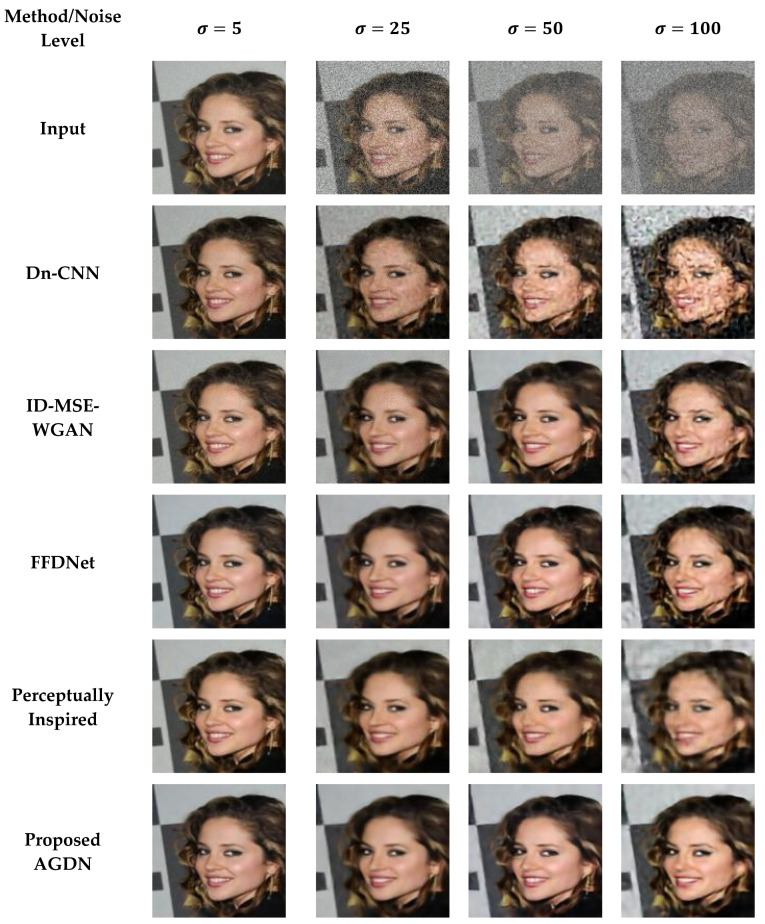
Third sample results of image denoising tasks on the Partial-CelebA dataset. The first to last column images generated by the noise level of sigma 5, 25, 50, and 100, respectively. First-row shows input images, second-row to the last-row presents the results of the Dn-CNN, ID-MSE-WGAN, FFDNet, the perceptually inspired method, and the proposed AGDN, respectively.

**Figure 10:**
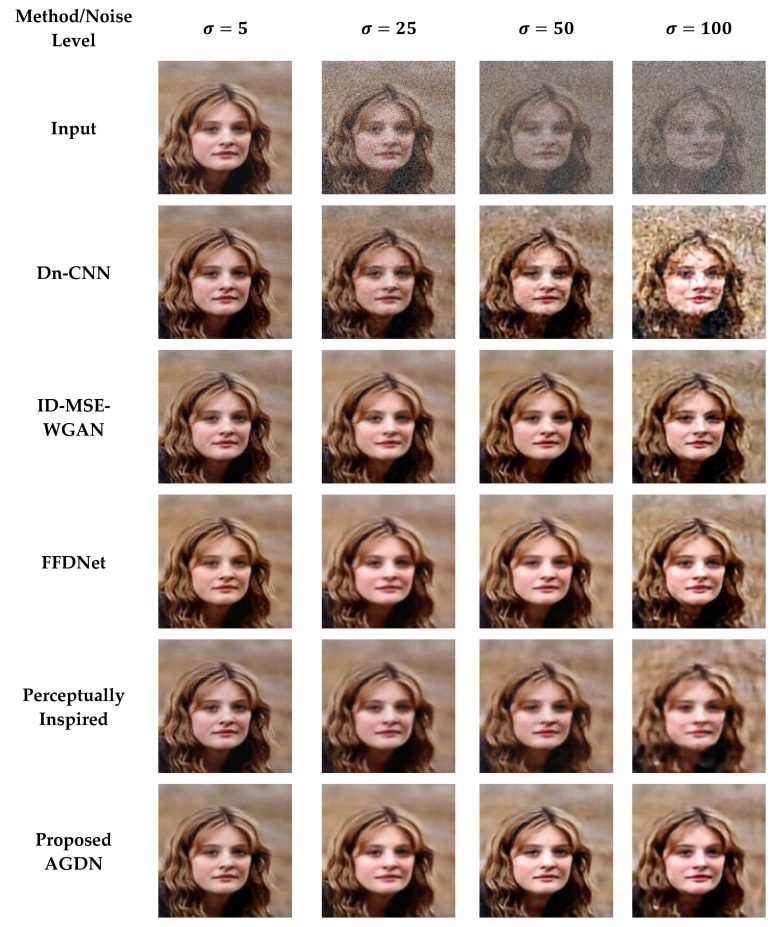
Fourth sample results of image denoising tasks on the Partial-CelebA dataset. The first to last column images generated by the noise level of sigma 5, 25, 50, and 100, respectively. First-row shows input images, second-row to the last-row presents the results of the Dn-CNN, ID-MSE-WGAN, FFDNet, the perceptually inspired method, and the proposed AGDN, respectively.

We attempt to denoise the AWGN’s multiple noise levels on the Partial-CelebA dataset, as shown in Figure 7. More examples are given in Figure 8, Figure 9 and Figure 10. The network is trained on 1500 images and tested on 500 images of the Partial-CelebA dataset for each noise level. We run tests on 500 test images for fair comparison and take the average to calculate quantitative scores of PSNR, SSIM, UQI, and VIF. Table 9 presents the quantitative comparison of state-of-the-art methods and the proposed method. Table 9 shows that for the noise level of sigma 5, the ID-MSE-WGAN, the perceptually inspired method, and the Dn-CNN achieve reasonably good scores in PSNR, SSIM, UQI, VIF, and FID. However, when the noise level increases, these methods fail to construct pleasing images. One possible reason is that the Dn-CNN and the ID-MSE-WGAN learn the noise image first instead of directly learning the target image. As the noise level increases, it becomes harder for the network to learn noise images first and then construct the target images compared to learning the target image directly.

Additionally, the perceptually inspired method fails because they use skip-connections in its denoiser network. The skip-connections cause the flow of unwanted information directly from the encoder layers to the decoder layers. When the noise level increases, there is more chance to transfer the noisy texture of the input images in the generated images. We observed from Figure 7, Figure 8, Figure 9 and Figure 10 that the FFDNet constructed reasonable images for the low noise levels, but produced image artifacts for high-level noise. The proposed method achieved excellent scores for the high noise level compared to the baseline methods. After the examination, we found that the proposed approach captured more content information and constructed sharp, artifact-free, and more similar clean images to the clean targeted images. Moreover, the quantitative comparison in Table 9 also describes that the proposed method achieved a high average score for all the noise levels in PSNR, SSIM, UQI, VIF, and FID, which means that the proposed method can significantly achieve improved results.

#### 4.6.2. DIV2K Dataset

**Figure 11:**
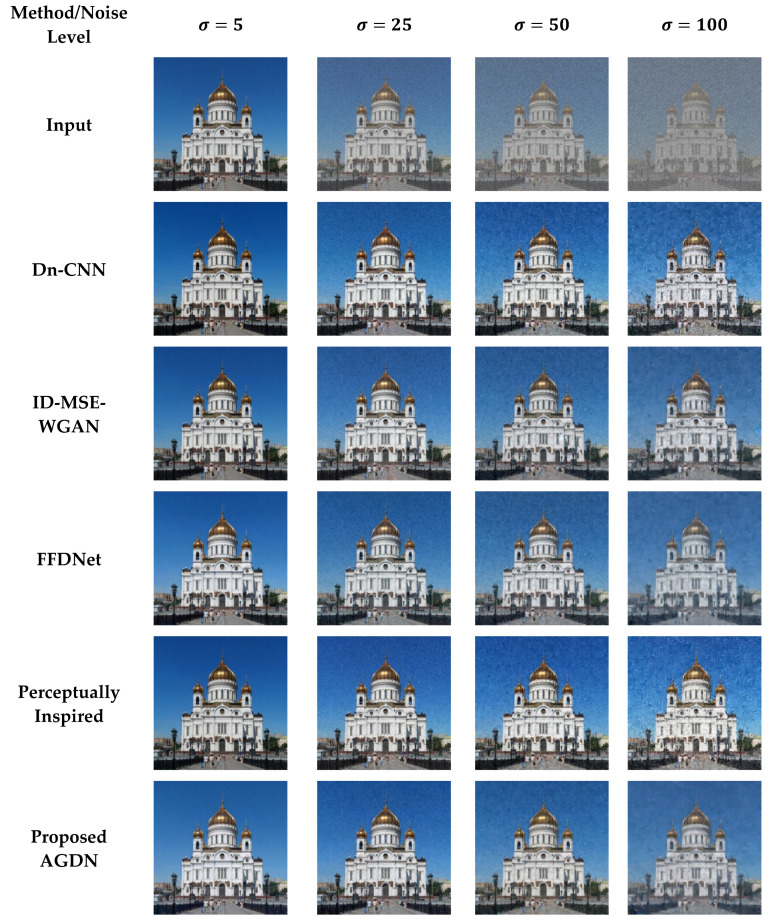
First example results of image denoising tasks on the DIV2K dataset. The first to last column images generated by the noise level of sigma 5, 25, 50, and 100, respectively. First-row shows input images, second-row to the last row presents the results of Dn-CNN, ID-MSE-WGAN, FFDNet, the perceptually inspired method, and the proposed AGDN, respectively.

**Figure 12:**
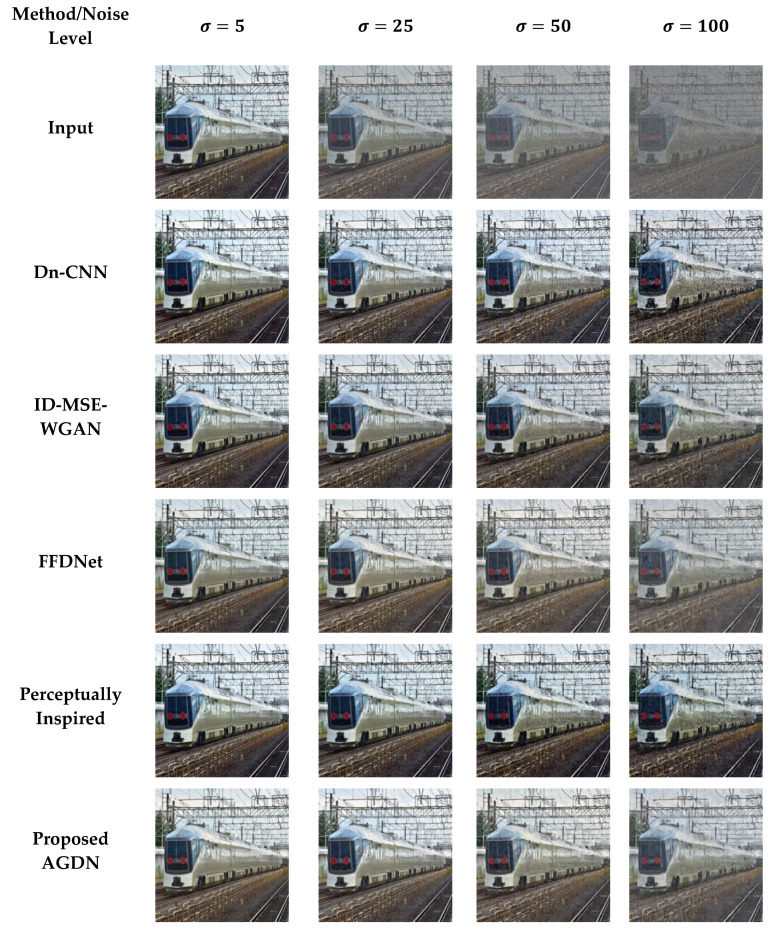
Second example results of image denoising tasks on the DIV2K dataset. The first to last column images generated by the noise level of sigma 5, 25, 50, and 100, respectively. First-row shows input images, second-row to the last row presents the results of Dn-CNN, ID-MSE-WGAN, FFDNet, the perceptually inspired method, and the proposed AGDN, respectively.

**Figure 13:**
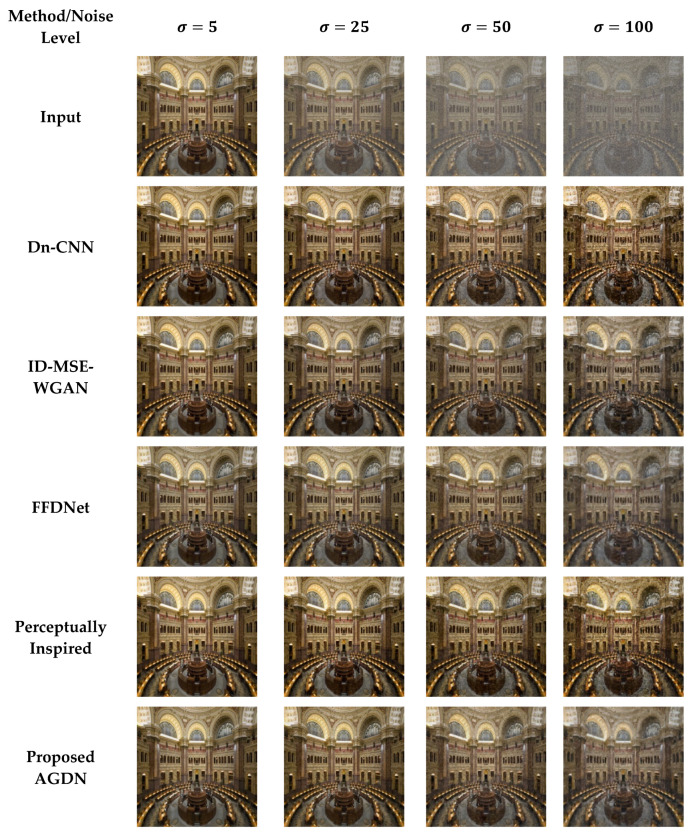
Third example results of image denoising tasks on the DIV2K dataset. The first to last column images generated by the noise level of sigma 5, 25, 50, and 100, respectively. First-row shows input images, second-row to the last row presents the results of Dn-CNN, ID-MSE-WGAN, FFDNet, the perceptually inspired method, and the proposed AGDN, respectively.

**Figure 14:**
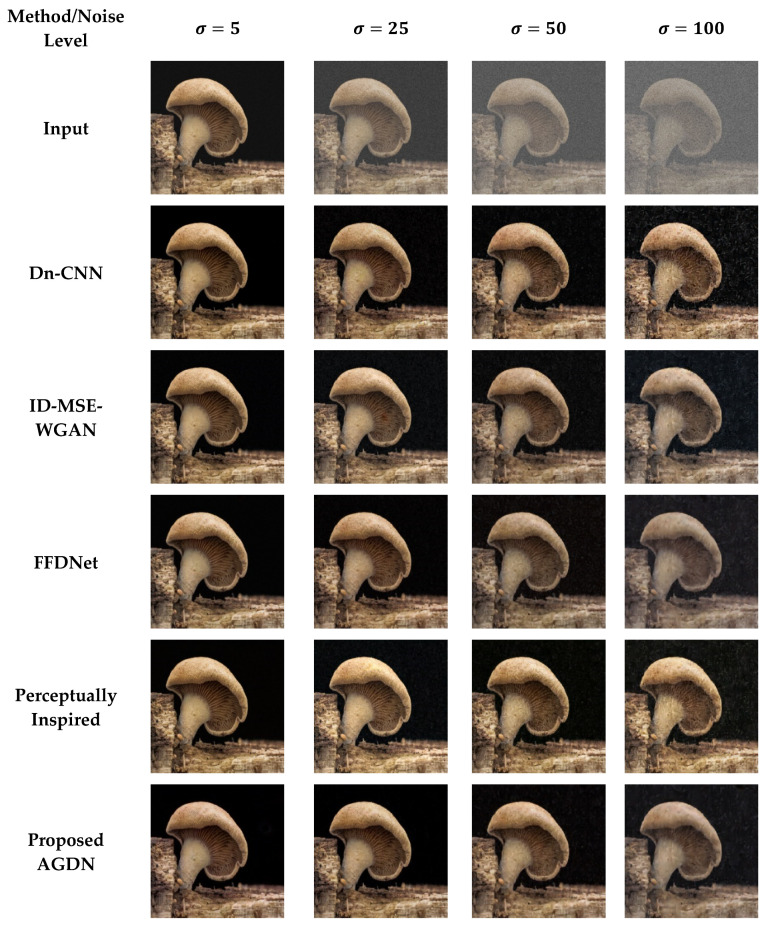
Fourth example results of image denoising tasks on the DIV2K dataset. The first to last column images generated by Table 5. 25, 50, and 100, respectively. First-row shows input images, second-row to the last row presents the results of Dn-CNN, ID-MSE-WGAN, FFDNet, the perceptually inspired method, and the proposed AGDN, respectively.

On the DIV2K dataset, we also intend to denoise the AWGN’s multiple noise levels, as shown in Figure 11, Figure 12, Figure 13 and Figure 14. The network was trained on 800 images and validated on 100 images of the DIV2K dataset for each noise level. We conducted tests on 100 test images and averaged the results to measure quantitative PSNR, SSIM, UQI, VIF, and FID score for a valid assessment. Table 10 provides a quantitative comparison of the proposed and state-of-the-art methods. Table 10 shows that for the low noise level sigma value of 5, the Dn-CNN achieved higher quantitative scores of PSNR, SSIM, UQI, and a reasonable score in FID. However, when the noise level increased, the Dn-CNN method obtained an inferior FID score, which showed that the constructed image domain was far from the targeted domain. One possible reason is that the Dn-CNN aims to learn the residual image first instead of directly learning the targeted image. When the noise level starts to rise, learning noise images first and then constructing target images becomes more difficult for the network than learning the target image directly.

Additionally, Figure 11, Figure 12, Figure 13 and Figure 14 show that the images generated by the perceptually inspired method contain more noise content compared to the proposed method at higher noise levels. The perceptually inspired method’s skip-connections cause the flow of unwanted information directly from the encoder layers to the decoder layers, hence containing more noise content in the constructed images at higher noise levels. However, the proposed method had an inferior performance to maintain the color content compared to the perceptually inspired method, but removed more noise content from the resultant image and remained closer to the actual structure content of the noise-free target image. Table 10 shows that the AGDN achieved the best possible PSNR, SSIM, UQI, VIF, and FID scores. Hence, the constructed noise-free images were more similar to the targeted noise-free images and had a recognizable structure, and were visually pleasing.

## 5. Conclusions

We introduced a robust image denoising scheme that was adversarial inspired and constructed sharp and visually pleasing images for all noise levels. This paper proposed a novel adversarial Gaussian denoiser network (AGDN) for image denoising tasks as a general-purpose framework for all noise levels. We merged the adversarial and the per-pixel Euclidean reconstruction losses as the state-of-the-art loss function for image denoising tasks. The proposed loss function helps our model to focus on target-oriented and fine image detail preservation. Additionally, we investigated two traditional image denoising methods (i.e., the residual learning and the direct image denoising methods) on two primary network configurations. We assessed their results qualitatively and quantitatively. The denoiser network without skip-connections constructed high quality and graphically pleasing clean images than a denoiser network with skip-connections for all noise levels. We conducted substantial experiments on lower and higher noise levels to evaluate the competence of the AGDN. The proposed method outperformed the current state-of-the-art methods for image denoising tasks. The experimental results of the multiple noise levels of image denoising tasks demonstrated that the adopted method is effective and capable of multiple practical levels of image denoising applications. We will look for a denoising approach for future work to manage real complex noise since this work focuses only on AWGN noise.

## Figures and Tables

**Table 1:** Comparison of the proposed and state-of-the-art methods.

Methods		Advantages	Disadvantages
Model-Based Methods	Priors of Sparsity [15,16], Non-local Self-Similarity (NSS) [17,18,19,20]	Advantage of handcrafted image priors Performed well for low noise levels	Limited for high noise levels The inference phase is time-consuming
Discriminative Learning-based methods	NOISE2NOISE [21], NOISE2VOID [22]	These methods can overcome the need to train a denoising network for many pairs of images.	Constrained by the prior information used
	Dn-CNN [23], ID-MSE-WGAN [45]	Fast inference time Performance is suitable for low noise levels	Produce image artifacts for higher levels of noise
	FFDNet [24]	Fast and flexible Capture larger receptive field by downsampling images	Can cause loss of important information by downsampling images
	The perceptually inspired denoising method [46],	Fast testing time Construction of denoised images perceptually strong for low noise levels Used skip-connections to maintain the content of the image	The skip-connections cause the flow of unwanted information to the output images and hence producing image artifacts for higher levels of noise
	The proposed AGDN	Fast inference time Construct sharp, pleasing, and target-oriented images for all levels of noise Used residual blocks for deeper network	Need to train extra discriminator Network Take slightly extra time during training

**Table 2:** Denoiser network of AGDN.

	Padding	Kernel Size	Operation	Feature Maps	Stride	Non-Linearity
Encoder	3	7	Convolution	64	1	ReLU
	1	3	Convolution	128	2	ReLU
	1	3	Convolution	256	2	ReLU
Residual Blocks	1	3	Residual block	256	1	ReLU
	1	3	Residual block	256	1	ReLU
	1	3	Residual block	256	1	ReLU
	1	3	Residual block	256	1	ReLU
	1	3	Residual block	256	1	ReLU
	1	3	Residual block	256	1	ReLU
	1	3	Residual block	256	1	ReLU
	1	3	Residual block	256	1	ReLU
	1	3	Residual block	256	1	ReLU
Decoder	1	3	Deconvolutional	128	2	ReLU
	1	3	Deconvolutional	256	2	ReLU
	3	7	Convolutional	256	1	Tanh

**Table 3:** Residual block network.

Padding	Kernel Size	Operation	Feature Maps	Stride	Non-Linearity	Dropout
1	3	Convolution	256	1	ReLU	0.5
1	3	Convolution	256	1	-	-

**Table 4:** Discriminator network.

Padding	Kernel Size	Operation	Feature Maps	Stride	Non-Linearity
1	4	Convolution	64	2	LeakyReLU
1	4	Convolution	128	2	LeakyReLU
1	4	Convolution	256	2	LeakyReLU
1	4	Convolution	512	1	LeakyReLU
1	4	Convolution	1	1	Sigmoid

**Table 5:** Quantitative results for the noise level of sigma 25 compared with different loss functions. Bold results show good scores.

	PSNR (dB)	SSIM	UQI	VIF	FID
Input	17.48	0.5725	0.8173	0.2384	106.4
L2	27.15	0.9142	0.9457	0.4765	44.99
L2 + Adv_loss	26.22	0.9028	0.9381	0.4546	48.07
L1	25.62	0.8924	0.9371	0.4355	51.49
Proposed	**27.41**	**0.9184**	**0.9483**	**0.4879**	**42.73**

**Table 6:** Quantitative results for the noise level of sigma 50 compared with different loss functions. Bold results show good scores.

	PSNR (dB)	SSIM	UQI	VIF	FID
Input	14.66	0.5570	0.7618	0.2089	131.0
L2	26.48	0.9130	0.9432	0.4835	43.41
L2 + Adv_loss	26.35	0.9082	0.9427	0.4801	45.31
L1	25.41	0.8858	0.9180	0.4257	53.64
Proposed	**26.54**	**0.9148**	**0.9437**	**0.4850**	**42.24**

**Table 7:** Quantitative results for the noise level of sigma 100 compared with different loss functions. Bold results show good scores.

	PSNR (dB)	SSIM	UQI	VIF	FID
Input	12.74	0.3792	0.7204	0.1104	252.9
L2	24.39	0.8503	0.9243	0.4259	80.11
L2 + Adv_loss	24.18	0.8442	0.9257	0.4169	84.82
L1	24.83	0.8840	**0.9261**	0.4421	59.53
Proposed	**24.90**	**0.8850**	0.9251	**0.4434**	**59.33**

**Table 8:** Quantitative results of different methods with primary two configurations of generating the model on several noise levels. Bold results show good scores.

Method/Noise Level	σ=5	σ=25	σ=50	σ=100	Average
**PSNR (dB)**					
U-NET-RLID	31.65	26.92	25.54	21.49	26.40
U-NET-DID	31.28	27.23	26.06	22.67	26.81
Enc-Dec-RLID	**31.67**	**27.45**	**26.85**	24.79	**27.69**
Enc-Dec-DID	28.75	25.62	25.40	24.83	26.15
Proposed AGDN	28.85	27.41	26.54	**24.90**	26.92
**SSIM**					
U-NET-RLID	**0.9638**	0.9097	0.8199	0.6502	0.8359
U-NET-DID	0.9605	0.9076	0.8734	0.7361	0.8694
Enc-Dec-RLID	0.9604	0.9014	**0.9165**	0.8482	0.9066
Enc-Dec-DID	0.9571	0.8924	0.8857	0.8840	0.9048
Proposed AGDN	0.9570	**0.9184**	0.9148	**0.8850**	**0.9188**
**UQI**					
U-NET-RLID	0.9650	0.9418	0.9306	0.8967	0.9335
U-NET-DID	0.9633	0.9459	0.9359	0.9122	0.9393
Enc-Dec-RLID	**0.9675**	**0.9488**	0.9366	**0.9266**	**0.9449**
Enc-Dec-DID	0.9557	0.9371	0.9179	0.9261	0.9342
Proposed AGDN	0.9565	0.9483	**0.9437**	0.9251	0.9434
**VIF**					
U-NET-RLID	**0.6275**	0.4750	0.3857	0.2560	0.4360
U-NET-DID	0.6186	0.4674	0.4183	0.2997	0.4510
Enc-Dec-RLID	0.6273	0.4735	**0.4906**	0.4233	0.5037
Enc-Dec-DID	0.6055	0.4355	0.4256	0.4421	0.4772
Proposed AGDN	0.6062	**0.4879**	0.4850	**0.4434**	**0.5056**
**FID**					
U-NET-RLID	20.55	45.34	79.58	133.8	69.82
U-NET-DID	20.86	44.65	61.60	107.5	58.65
Enc-Dec-RLID	**19.77**	46.63	**42.26**	88.91	49.39
Enc-Dec-DID	23.40	51.49	53.64	59.53	47.01
Proposed AGDN	23.79	**42.73**	44.24	**59.33**	**42.52**

**Table 9:** Quantitative results of baseline methods with the proposed method on several noise levels. Bold results show good scores.

Method/Noise Level	σ=5	σ=25	σ=50	σ=100	Average
**PSNR (dB)**					
Noisy Input	28.64	17.48	14.66	12.74	18.38
Dn-CNN	31.23	25.78	24.77	20.26	25.51
ID-MSE-WGAN	**31.30**	26.12	25.15	24.23	26.70
FFDNet	28.66	26.95	26.30	24.39	26.58
Perceptually Inspired	31.28	26.63	25.86	22.37	26.53
Proposed AGDN	28.85	**27.41**	**26.54**	**24.90**	**26.92**
**SSIM**					
Noisy Input	0.9653	0.5725	0.5570	0.3792	0.6185
Dn-CNN	0.9594	0.8554	0.7805	0.5862	0.7953
ID-MSE-WGAN	**0.9657**	0.9020	0.8890	0.8492	0.9014
FFDNet	0.9583	0.9130	0.9120	0.8503	0.9084
Perceptually Inspired	0.9605	0.9076	0.8734	0.7361	0.8694
Proposed AGDN	0.9570	**0.9184**	**0.9148**	**0.8850**	**0.9188**
**UQI**					
Noisy Input	0.9265	0.8173	0.7618	0.7204	0.8065
Dn-CNN	0.9672	0.9390	0.9265	0.8804	0.9282
ID-MSE-WGAN	**0.9677**	0.9358	0.9261	0.9276	0.9393
FFDNet	0.9555	0.9480	0.9429	0.9243	0.9426
Perceptually Inspired	0.9633	0.9459	0.9359	0.9122	0.9393
Proposed AGDN	0.9565	**0.9483**	**0.9437**	**0.9251**	**0.9434**
**VIF**					
Noisy Input	0.7016	0.2384	0.2089	0.1104	0.3148
Dn-CNN	0.6221	0.4258	0.3546	0.2232	0.4064
ID-MSE-WGAN	**0.6275**	0.4605	0.4801	0.4243	0.4981
FFDNet	0.6123	0.4821	**0.4972**	0.4249	0.5041
Perceptually Inspired	0.6186	0.4674	0.4183	0.2997	0.4510
Proposed AGDN	0.6062	**0.4879**	0.4850	**0.4434**	**0.5056**
**FID**					
Noisy Input	19.78	106.43	131.0	252.9	127.5
Dn-CNN	20.46	88.12	124.2	228.5	115.3
ID-MSE-WGAN	**19.70**	47.93	45.62	87.61	50.21
FFDNet	22.03	42.99	44.40	80.11	47.38
Perceptually Inspired	20.86	44.65	61.60	107.5	58.65
Proposed AGDN	23.79	**42.73**	**44.24**	**59.33**	**42.52**

**Table 10:** Quantitative results of baseline methods with the proposed method on the DIV2K dataset of multiple noise levels. Bold results show good scores.

Method/Noise Level	σ=5	σ=25	σ=50	σ=100	Average
**PSNR (dB)**					
Noisy Input	29.57	18.09	15.00	13.14	18.95
Dn-CNN	**33.32**	25.61	22.92	20.66	25.62
ID-MSE-WGAN	32.50	25.27	22.71	20.43	25.23
FFDNet	25.90	24.39	21.97	19.15	22.85
Perceptually Inspired	32.54	**26.27**	23.41	21.15	25.84
Proposed AGDN	32.88	26.01	**23.48**	**21.52**	**25.97**
**SSIM**					
Noisy Input	0.9521	0.7248	0.5399	0.3612	0.6445
Dn-CNN	**0.9719**	0.8599	0.7683	0.6217	0.8055
ID-MSE-WGAN	0.9670	0.8466	0.7573	0.6347	0.8014
FFDNet	0.9294	0.8618	0.7786	0.6637	0.8084
Perceptually Inspired	0.9643	0.8653	0.7868	0.6725	0.8222
Proposed AGDN	0.9673	**0.8709**	**0.7897**	**0.6815**	**0.8274**
**UQI**					
Noisy Input	0.9508	0.8382	0.7825	0.7440	0.8289
Dn-CNN	**0.9740**	**0.9493**	**0.9312**	0.9008	0.9388
ID-MSE-WGAN	0.9707	0.9340	0.9164	0.8973	0.9296
FFDNet	0.9362	0.9271	0.9046	0.8715	0.9099
Perceptually Inspired	0.9686	0.9414	0.9295	0.9112	0.9377
Proposed AGDN	0.9715	0.9472	0.9285	**0.9135**	**0.9402**
**VIF**					
Noisy Input	**0.7931**	0.4121	0.2500	0.1272	0.3956
Dn-CNN	0.7851	0.4638	0.3390	0.2219	0.4525
ID-MSE-WGAN	0.7889	0.4655	0.3361	0.2263	0.4542
FFDNet	0.6253	0.4615	0.3458	0.2491	0.4204
Perceptually Inspired	0.7598	**0.4787**	0.3516	0.2408	0.4577
Proposed AGDN	0.7890	0.4696	**0.3479**	**0.2524**	**0.4647**
**FID**					
Noisy Input	11.33	87.80	157.0	225.1	120.3
Dn-CNN	11.63	70.64	120.4	179.1	95.44
ID-MSE-WGAN	**11.21**	**67.80**	118.9	174.3	93.05
FFDNet	24.52	69.06	108.0	152.5	88.52
Perceptually Inspired	12.90	68.73	112.8	158.2	88.16
Proposed AGDN	15.11	68.29	**107.1**	**150.7**	**85.30**

## Data Availability

The datasets used in this work are available online with open access for non-commercial academic research use only. The Partial-CelebA dataset is available online at http://mmlab.ie.cuhk.edu.hk/projects/CelebA.html (Accessed 15 February 2021), and the DIV2K dataset is available online at https://data.vision.ee.ethz.ch/cvl/DIV2K/ (Accessed 15 February 2021).

## Abbreviations

AWGN	Additive White Gaussian Noise
NSS	Non-Local Self-Similarity
BM3D	Block-Matching and 3D filtering
CNNs	Convolutional Neural Networks
Dn-CNN	Feedforward Denoising CNN
FFDNet	Fast and Flexible Denoising Network
GANs	Generative Adversarial Networks
cGANs	Conditional Generative Adversarial Networks
WGANs	Wasserstein Generative Adversarial Networks
AGDN	Adversarial Gaussian Denoiser Network
MRF	Markov Random-Field
NLR-MRF	Non-Local Range MRF
MAP	Maximum A Posteriori
CSF	Cascade of Shrinkage Fields
TNRD	Trainable Nonlinear Reaction-Diffusion
PSNR	Peak Signal to Noise Ratio
SSIM	Structural Similarity Index Measurement
VIF	Visual Information Fidelity
UQI	Universal Quality Index
FID	Fréchet Inception Distance
RLID	Residual Learning Image Denoising
DID	Direct Image Denoising
